# Strengthening the ethical assessment of placebo-controlled surgical trials: three proposals

**DOI:** 10.1186/1472-6939-15-78

**Published:** 2014-10-23

**Authors:** Wendy Rogers, Katrina Hutchison, Zoë C Skea, Marion K Campbell

**Affiliations:** Department of Philosophy and Australian School of Advanced Medicine, Macquarie University, Sydney, NSW 2109 Australia; Department of Philosophy, Macquarie University, Sydney, NSW 2109 Australia; Health Services Research Unit, University of Aberdeen, Aberdeen, AB25 2ZD UK

**Keywords:** Placebo-controlled surgical trials, Research ethics, Therapeutic misconception, Research methods, Surgical research, Efficacy, Placebo effect, Consumer involvement

## Abstract

**Background:**

Placebo-controlled surgical trials can provide important information about the efficacy of surgical interventions. However, they are ethically contentious as placebo surgery entails the risk of harms to recipients, such as pain, scarring or anaesthetic misadventure. This has led to claims that placebo-controlled surgical trials are inherently unethical. On the other hand, without placebo-controlled surgical trials, it may be impossible to know whether an apparent benefit from surgery is due to the intervention itself or to the placebo effect.

**Discussion:**

In this paper we investigate justifications for placebo-controlled surgical trials and suggest three measures for strengthening their ethical acceptability. We argue that, given the extent, irreversibility and cost of surgical interventions, there is a need for the best possible evidence about their efficacy. In some cases, the strongest evidence will be from placebo-controlled surgical trials, especially where interventions are for outcomes (such as pain) that are likely to elicit a placebo response. In the second part of the paper, we propose three specific measures to increase the ethical acceptability of placebo-controlled surgical trials. The first is structured consultation with the relevant patient community about the risks and benefits of particular placebo-controlled surgical trials. The second seeks to address the therapeutic misconception through the use of educational materials, informed by patient consultation. Finally, we argue for ethical consideration of non-surgeon clinicians who are necessarily involved in the delivery of placebo-surgical interventions.

**Summary:**

If there is no appropriate surgical comparator and the risks can be reduced to the absolute minimum (given the type of placebo procedure required), and the research has the support of the relevant patient community, there may be grounds for judging that the potential benefits of specific placebo-controlled surgical trials outweigh the risks. If so justified, the ethical acceptability of placebo-controlled surgical trials can be enhanced through using educational measures to address participant vulnerability, and by recognizing clinicians who are necessary participants in the research.

## Background

With surgery, as with other branches of health care, it is desirable to have evidence that the intervention in question works (achieves the intended outcomes) before offering it to patients. Otherwise, patients may be exposed to invasive and harmful procedures for little or no benefit. Clinical trials measure efficacy by testing the intervention against a comparator of known efficacy, or where there is no suitable comparator, against a placebo. The most rigorous clinical trials blind both clinicians and participants as to who is receiving the intervention in question; so that any effects may be attributed to the intervention itself. In surgical research where there is no suitable comparator, proving efficacy may require the use of placebos. This entails exposing participants to a placebo or sham operation, with potential risk of harms. Placebos are procedures that are considered to be ‘inert’ [1]. In surgery, a placebo mimics the surgical intervention but without the intended therapeutic procedure. Surgical placebos vary in their degree of invasiveness. Some may avoid general anaesthesia and use incisions that penetrate the epidermis only [2] while others involve anaesthesia, and create surgical wounds leading to pain and scarring [3]. As surgical placebos need to be plausible, they raise serious questions about the potential levels of harm to which trial participants may be exposed for the purposes of research.

The issue of knowingly exposing participants in placebo-controlled surgical trials (PCSTs) to the harms of a sham procedure has polarised debate about their ethical acceptability [4–8]. The debate has been framed as a clash between the highest standards of research design and the highest standards of research ethics [8].

Proponents of PCSTs argue that these trials are sometimes necessary to find out whether particular surgical interventions are efficacious, or whether any observed changes in a patient’s condition are due to a placebo effect [3, 7, 9, 10].^a^ The requirement to minimise the risk of harms is understood to be ethically important, but not one that necessarily overrides the requirement for research to be of the highest scientific standards [7, 11, 12]. Taking this view, researchers are obliged to minimise the risks consistent with using the most appropriate trial design [13], which in surgery, may be a PCST in order to answer questions about efficacy. Without such answers, surgical patients, sometimes many thousands, are potentially exposed to surgical interventions that may be ineffective – described by one surgeon as no better than “glorified acupuncture” [14]. A trial that identifies a surgical intervention as no better than placebo has significant implications for practice. For example, Sihvonen et al. [15] estimate that upward of 700,000 patients in the US undergo arthroscopic partial meniscectomies each year, yet their trial shows the procedure to be no better than placebo across a range of outcomes. Those supportive of PCSTs acknowledge that participants may be harmed by receiving a sham procedure, but claim that the risk of harm (in at least some trials) can be minimized to the extent that it becomes reasonable to make a judgment about the balance between these risks and the value to society of the potential knowledge to be gained about the intervention’s efficacy. On their account, ethical bodies responsible for reviewing research (institutional review boards – IRBs) should make this judgment and approve methodologically rigorous PCSTs in which the risk of harms have been minimised, leaving the ultimate decision as to whether the risks of placebo trial participation are worthwhile to prospective participants, duly protected by adequate informed consent processes.

Conversely, opponents of PCSTs claim that placebo surgery is inherently harmful and therefore breaches the ethical obligations of researchers to minimise risk of harm to participants [5, 6, 16]. On this view, *any* risk of harm to individuals is unacceptable. Notwithstanding that PCSTs will yield information about the efficacy of surgical interventions that is otherwise unobtainable, for Macklin, “performing a surgical procedure that has no expected benefit other than the placebo effect violates the ethical and regulatory principle that the risk of harm to subjects must be minimized in the conduct of research” [6], p. 993. Macklin and others [5, 16] argue that the ethical injunction to minimize the risk of harms must take precedence over any likely gains to knowledge, no matter how great their promise. These authors argue that informed consent is inadequate to protect participants. In their view, IRBs should hesitate to approve such trials, irrespective of their scientific merit.

## Discussion

In this paper, we investigate assumptions that ground claims about the necessity of placebo-controlled surgical trials and make suggestions for three measures that may be of use to IRBs charged with reviewing PCST protocols. First we argue that there is a need for reliable and valid evidence about surgical interventions in view of their extent, irreversibility and cost. We present evidence for the existence of the surgical placebo effect and argue that given the placebo response, PCSTs are necessary, in cases where there is no appropriate surgical comparator, to provide robust evidence about the efficacy of surgical interventions that is not otherwise obtainable. In the second part of the paper, we propose three specific measures to increase the ethical acceptability of PCSTs. First, we argue that structured consultation with the relevant patient community might provide valuable information to IRBs weighing up the risks and benefits of particular PCSTs. Second, we advocate the use of educational materials, informed by patient consultation, to mitigate as far as possible the therapeutic misconception. Third, we argue that, given the risks to which they are exposed, formal agreement should be sought from non-surgeon clinicians who are necessarily involved in the surgical intervention. This paper draws on previous qualitative research undertaken by two of the authors into the design, acceptability and feasibility of PCSTs [14, 17].

### Is there a need for placebo-controlled surgical trials?

Placebo-controlled surgical trials entail the risk of harms to participants, and therefore warrant careful analysis of their alleged necessity. Here we argue that surgery is an important and costly health care intervention, and as such, should be backed by the strongest possible evidence base, which at times, includes that generated by PCSTs. Surgery plays an increasing role in modern health care. It has been estimated that people living in industrialised countries will have five surgical procedures during their lifetimes [18]. In Australia in 2011–12, there were nearly two and a half million hospital discharges following surgical procedures, an increase of 3.6% over the previous year [19], p. 211. The increase in surgical procedures may reflect increased longevity leading to greater numbers of people living with age-related degenerative disorders and cancers amenable to surgical intervention, or the increasing scope of surgery, for example in the treatment of weight management. Irrespective of the specific causes, rates of surgical interventions are on the increase [19].

As rates of surgery rise, so do the costs. These may be viewed in two ways – health care costs to society of surgical procedures; and costs to patients in terms of pain and suffering. For patients, surgical procedures involve significant disruptions to daily life, including admission to hospital (in 2010–11, Australian patients spent a total of 1,653,474 days in hospital for procedures related to musculoskeletal conditions alone [20], p. 256). Surgery commonly requires some kind of anaesthesia, which entails risks over and above those of the surgical intervention; and the chance of complications such as infection or excessive blood loss. A period of convalescence is usually required following surgery, during which patients may experience pain, have decreased mobility and generally be unable to live their normal lives. Finally, surgery is largely irreversible. It may be possible to remove a surgical prosthesis, but it is not possible to ‘undo’ an operation: surgery leaves permanent traces on and in the bodies of patients.

In addition to these real and potential harms to patients, surgery can be expensive. The 700,000 annual arthroscopic partial meniscectomies mentioned above incur annual direct medical costs of US$4 billion [15]. Healthcare costs are rising around the world. In all OECD countries (apart from Finland), health expenditure as a proportion of GDP rose between 2001 and 2011 [21]. In Australia, for example, expenditure rose from AUD$63,099 million in 2002 to AUD$140,241 million in 2013 [21]. Given that rates of surgical interventions are increasing, it is fair to consider that at least a proportion of these rises in healthcare expenditure are driven by surgery. Rising costs may of course be justified if increases in expenditure lead to better health outcomes. And clearly some surgical interventions are highly cost effective. For example, kidney transplantation is more effective and less costly than renal dialysis [22]. Nonetheless, in order to fund the ‘right’ interventions, there is a need for research capable of discerning between those interventions that are effective and those that are no better than placebo, as the latter are potentially both harmful and wasteful [9, 12].

This need for evidence of efficacy does not automatically translate into a need for PCSTs unless there are reasons to believe that PCSTs are required for developing a body of evidence sufficient to justify the surgical intervention in question. Placebo surgery may be unwarranted for two different sorts of reasons. In some cases, the new intervention can be compared directly with an existing surgical treatment that is known to be effective and safe, in which case a randomized controlled trial does not need a placebo arm. In other cases placebo surgery is unwarranted because the patient’s condition requires some surgical intervention to avert a potentially catastrophic outcome. For example, it would not be warranted to do a PCST for ruptured cerebral aneurysms, or appendicitis, as it would not be ethical to allocate participants to a placebo given that effective treatments for these conditions exist, and without some intervention, the patient is at unacceptably high risk of an adverse outcome.

The strength of PCSTs is that they have the potential to disentangle the causal effects of the surgical intervention from any potential placebo effects. Here we must clarify exactly what work the placebo performs in PCSTs, for as London and Kadane note, there are two meanings for the term “placebo effect” [5]. The first is methodological, where placebos are used to help control for as many variables as possible in a research trial, so that any effects of the active intervention may be attributed to that intervention rather than to other causes, such as the act of being admitted to hospital or anaesthetised. Here the placebo has an epistemic function – to sort out causal from other variables [23]. Second, placebos may have a therapeutic effect. This refers to the phenomenon of a person experiencing beneficial effects from an intervention which is provided in a therapeutic guise but which is deemed by health care providers to lack specific therapeutic powers, be these pharmacological, surgical or other [24]. In PCSTs, placebos control for both the therapeutic placebo effect of the intervention, and also serve an epistemic role to control for all other non-specific effects such as the natural course of the disease or regression to the mean. However, we believe PCSTs are of value only to the extent that there are good reasons to believe that there is a placebo effect in surgery, as if there is not, PCSTs are never justified.^b^

In addressing this point, we seek to provide a solid foundation for the work of other authors [8, 12, 25] who have proposed criteria that should be met in order to justify performing specific PCSTs^c^; our aim is to examine the assumption about the placebo effect that underlies their support for PCSTs. We now turn to evidence for the existence of a surgical placebo effect.

Beecher’s 1961 paper on surgery as placebo is foundational in this regard [26]. Beecher analysed the results of a number of trials of internal mammary artery ligation for severe angina. The trials are of varying methodological quality, but include two randomised placebo-controlled studies [27, 28]. Beecher discusses the remarkable recovery of one patient in the Cobb et al. trial who, after receiving the placebo operation, increased his exercise tolerance from 4 to 10 minutes and reversed the ECG signs of ischemia that previously occurred within 4 minutes. As Beecher notes, “It is of such “convincing” objective stuff that new operations are made” [26], p. 1104. Individual trials published since that time also demonstrate empirical evidence of placebo effects following surgical interventions [2, 15, 26].

A systematic review of the placebo effect in eight surgical trials found that between 30-100% of participants in the placebo groups showed improvements in outcomes including pain, exercise tolerance, and dizziness, thereby supporting the claim that surgery appears to be associated with large placebo effects [29]. PCSTs seemed able to distinguish between effective and ineffective surgical interventions, in that of the eight trials included in the review, six showed no difference between placebo and a range of surgical interventions, and two showed that active surgery was more effective than placebo. Like the results of individual PCSTs, this review provides grounds for accepting that there is a surgical placebo effect.

Despite this evidence, the existence and importance of a surgical placebo effect has been disputed [30]. Hróbjartsson and Gøtzsche argue that Beecher and others calculate the placebo effect as change against baseline for the group receiving the placebo intervention, but this does not allow researchers to distinguish between other factors that might cause an apparent improvement including the natural course of the disease, or regression to the mean. They claim, “The reported large effects of placebo could therefore, at least in part, be artefacts of inadequate research methods” [31], p. 1594. However, the recent PCST by Sihvonen et al. [15] includes a separate publication with a detailed description of the study protocol that explicitly addresses these kinds of methodological criticisms [32]. Thus while it is possible that poorly designed PCSTs may not isolate the placebo effect from other causes of improvements, this criticism is not valid for all PCSTs demonstrating a placebo effect, and does not undermine the general claim that there is a surgical placebo effect.

In their review, Hróbjartsson and Gøtzsche make an important distinction concerning the kinds of outcomes where placebo effects are observed [31]. They conclude that there is little evidence that placebos have powerful clinical effects, where outcomes are binary (i.e. present or absent), or continuous and objectively assessed, such as physiological variables measured by health professionals. In contrast, where outcomes are subjectively assessed, and especially if continuous rather than binary, they found a significant placebo effect when compared with no treatment. Notably, pain is the outcome most powerfully affected by placebo interventions. This point is relevant for PCSTs as surgery is increasingly being used for elective and quality of life interventions with subjective outcomes, thus for interventions in which the placebo effect is likely to be most evident [9].

Given these findings, which have been consistent over time, it is reasonable to accept that surgery does exert a placebo effect, especially where the relevant outcomes are subjective and continuous, such as relief from symptoms or impact on quality of life [33]. This creates a prima facie justification for PCSTs to assess the efficacy of interventions designed to achieve these outcomes, because well-designed PCSTs can demonstrate whether or not the intervention is more effective than the placebo in causing observed changes in outcomes.

Another line of criticism accepts that while there may be an in-principle justification for PCSTs, specific instances of PCSTs are unjustified [30, 34]. Polgar and Ng claim that the trials of both foetal tissue implants for Parkinson’s Disease [3] and the arthroscopy trial [2] were unjustified as the information they yielded could have been obtained in other ways. For example, they write, “It is evident that arthroscopy was not an effective treatment for osteoarthritis because the improvements on key outcome measures were less than the ‘minimal important’ standards specified in Moseley et al. (2002). The conclusion was evident from the outcomes for the two treatment groups without reference to the results of the placebo controls” [30], p. 293. However, this criticism seems to miss the mark as the point that Moseley et al. make from their trial is that the active treatment was not better than placebo, therefore any observed effects were likely due to placebo rather than the active surgery. As with other critics [31], the points raised by Polgar and Ng are not sufficient to reject all PCSTs, and can be addressed by methodological rigour in at least some cases.

One important aspect of methodological rigour concerns the placebo itself, which, to be appropriate, must maximise the mimic whilst minimising the risk. The aim is for patients to receive a placebo intervention that is similar enough to the active intervention to be convincing, but omits any known therapeutic aspects [13]. In order to fulfil the epistemic function that is sought in PCSTs, the placebo intervention must lack what are thought to be the therapeutic parts of the procedure; the placebo must be inert, insofar as this is possible. Birch argues that surgical placebos are never inert, because any surgical wounding triggers local and systemic physiological responses that may influence the body’s response to the index condition and thereby elicit a biologically mediated improvement [35]. It is not clear what kind of evidence could resolve this question. Surgical placebos are clearly likely to have greater biological effects compared with pharmaceutical placebos such as sugar pills, but even the latter are not completely inert given that the body is active in the process of ingestion and digestion of the pill. Nevertheless, given that PCSTs have convincingly distinguished between effective and ineffective surgical interventions [26, 36], it seems warranted to assume that they are inert enough to fulfil their epistemic function in PCSTs.

Surgical placebos also must be convincing enough to prevent participants from guessing which intervention they have received [13]. This seems to be an achievable goal: there are examples in the literature of plausible mimics, in that patients and those assessing outcomes have been unable to tell whether or not an individual patient received the therapeutically-intended intervention or the placebo [14, 15, 17, 33].

In this section we argued for the desirability of evidence about the efficacy of surgical interventions, given their individual and public costs, and the role of PCSTs in providing this evidence so long as claims about the existence of the placebo effect of surgery are warranted. There is consistent evidence for the existence and importance of a surgical placebo effect, thereby justifying a requirement for PCSTs for at least some interventions, especially where these aim to alleviate pain or improve quality of life. Nonetheless, PCSTs entail balancing the present exposure of individual participants to risk of surgical harms against the knowledge gains and likely benefit to future patients and the wider population. In the next section we make three suggestions for strengthening the ethical acceptability of PCSTs by consulting the relevant patient population about acceptable risks and benefits; developing educational materials to address the therapeutic misconception; and taking account of the roles of non-surgeon clinicians in PCSTs.

### Three proposals to strengthen the ethical acceptability of PCSTs

There are two groups who are rarely included in any formal way in debates about which PCSTs are ethically justifiable. The first group is that of the patient population for whom the intervention may be indicated, and who will therefore be affected by the outcome of the trial; and the second are the non-surgeon clinicians involved in delivering the intervention or placebo. We suggest structured engagement with both groups in order to strengthen the ethical acceptability of methodologically justified PCSTs. Consultation with patients will help to address concerns about the risk-benefit balance of the trial and the adequacy of informed consent, while consultations with non-surgeon clinicians accords appropriate respect to those who necessarily become involved in the research as de facto collaborators if a PCST goes ahead, and who thus may be asked to engage in deception or expose themselves to professional risks.

Patients’ views can inform ethical deliberation about PCSTs in important ways [37]. For interventions to treat chronic conditions, those who are potential recipients of the proposed intervention are, through personal experience, generally well-informed about the nature of the condition and the strengths and weaknesses of current treatments. The affected group has a particular interest in understanding what is proposed and how it might affect them. This patient-held information is relevant for weighing up the risks and benefits of research as this is the cohort to whom any benefits will accrue (broadly understood, as the research will benefit future patients rather than the specific individuals consulted) if the intervention proves successful, and it is from this group that participants will be drawn if the research goes ahead. Patients affected by the index condition have a thorough understanding of the potential impact should the experimental intervention prove successful [17]. They are also the ones who would potentially undergo ineffective treatment if such a procedure became routine without rigorous trial. Their knowledge and experience are therefore highly relevant in weighing up risks and benefits of specific PCSTs. If members of the affected patient group consider that the potential benefits are such that at least some of them would consider participating in a PCST, this is relevant information for IRBs to take into account in their deliberations.^d^ IRBs that prohibit PCSTs remove the option of research participation from those who stand to benefit from the results of the research, whether or not the intervention itself is successful. On the other hand, IRBs that permit PCSTs without relevant consultation risk approving potentially harmful research, the outcomes of which are not viewed as valuable to patients.

In our view, deliberations about the acceptability of individual PCSTs should be informed by views from the relevant patient cohort about the risks and benefits of the research to them. This requires researchers to consult with patient groups and synthesise and present evidence about their views as part of their IRB application. Any consultation should be performed in a structured and rigorous way using appropriate qualitative methodology. This process of patient consultation will serve two purposes as it will address feasibility requirements (for example if the placebo is unacceptable, recruitment is unlikely to succeed) as well as inform ethical deliberation. We note that this may be seen as unduly burdensome – performing “pre-research” to develop the IRB application. However, there is increasing advocacy for co-production of research (research developed with potential participants) [38], some of which, such as the Peninsula Cerebral Research Unit in the UK, have active programs for including patients and their families in the generation of research questions.^e^ Our suggestion sits well with these developing standards. In some circumstances it may be desirable for national or international bodies to perform some of this preliminary work, ready for uptake by research teams. Finally, it is possible for funding bodies to release funds in a staged manner, which will allow for the necessary evidence to be gathered prior to moving to full funding of the proposal. There is no doubt that PCSTs are complex and challenging to implement but we do not believe that adding a requirement for consultation with patient groups will render them unmanageable. Furthermore, such pre-research may save considerable money and time if it averts full-blown trials of interventions that are unacceptable to members of the participant group, or likely to fail due to problems with recruitment.

Pre-research consultation has been used successfully for other controversial clinical trials. For example, Marsden and Bradburn report on the role of consultation with patient groups in assessing the acceptability of a trial of hormone replacement therapy (HRT) in women with breast cancer, which entails risks related to the oestrogen-dependence of most breast cancers [39]. Along similar lines, Koops and Lindley report on the role of focus groups with patients in confirming the acceptability of a trial for a high-risk stroke treatment. In this case, the pre-research with patients led to changes to patient information leaflets, and helped in terms of gaining IRB approval for the trial [14, 40].

Our second suggestion for improving the ethical acceptability of PCSTs is to develop materials to address the concern that informed consent is an inadequate protection because participants in PCSTs are vulnerable to the therapeutic misconception. This is the phenomenon that, despite explanations about trial design and randomisation, patients believe that their clinician/researcher will act in their best interests, and that the trial is a form of therapy [41]. There is evidence to suggest that participants do not fully appreciate the processes of clinical research [42, 43]. Studies have shown that patients misunderstand the nature of research including the randomisation process, and tend to believe that their doctor will act in their best interests, notwithstanding their enrolment into a trial [44, 45].

We agree that patients/participants are vulnerable to the therapeutic misconception and that this is a potential ethical challenge for PCSTs. However we disagree that the most appropriate remedy for this vulnerability is for IRBs to prohibit PCSTs. It may be ethically more appropriate to foster the capacity of potential participants better to understand the nature of clinical research and the dual role of the clinician/researcher. At the moment, IRBs have the options of prohibiting the trial or relying on individual informed consent to address any misconceptions. Neither of these options is ideal in terms of addressing the relevant vulnerability [46, 47]. The prohibition response is protective but does nothing to foster or support the understanding or decision-making capacity of the patients/participants. Additionally, routinely prohibiting PCSTs may leave patients worse-off. If the trial does not go ahead and the intervention therefore does not enter clinical practice, they are left with one less potential option for treatment. If the intervention is introduced into practice without proof of efficacy, they are at risk of receiving a treatment option that may not provide any benefit over placebo, or may be harmful.

The autonomy-respecting option of relying upon informed consent seems to deny any vulnerability on the part of patients/participants, and is known to be inadequate in many circumstances: the therapeutic misconception is certainly not limited to PCSTs [44]. We propose that one ethically appropriate way to address participant vulnerability to misconceptions is to draw upon the expertise of patient groups in developing educational materials about the trial. This could be achieved in a two step process: the first step would be part of the patient cohort consultation described above. As well as canvassing views about harms and benefits, the consultation could also identify issues likely to require clarification. In the second step, educational materials with information about the trial could be tested and refined if necessary with focus groups. The materials would specifically address likely misconceptions associated with a particular trial with the relevant patient group, along the lines of patient decision aids which have been developed to help people deliberate over and make treatment decisions [48]. Such materials could be designed for use in a public way via patient networks, well before trial enrolment commences, as well as during the informed consent process. The aim of these materials would be to increase research literacy, specifically address therapeutic misconceptions about the research trial for the particular intervention under investigation, and foster the decision making capacities of patients. This response provides a more nuanced and ethically justifiable way of addressing potential vulnerability rather than a blanket prohibition by IRBs [46].

This approach is supported by existing empirical work which suggests that patients are able to consider the risks and benefits of PCSTs, decide whether or not participation would be personally acceptable, and develop views about whether or not IRBs should approve the trials. Frank et al. surveyed three patient cohorts about the ethical acceptability of two hypothetical trials (one open, one blinded using placebo surgery) of a gene transfer agent for Parkinson’s Disease (PD). The three cohorts were patients with PD, patients with neurological conditions other than PD, and patients attending a community-based general internal medicine practice. Overall, patients with PD were less willing to participate in either trial than those without PD, but despite this, 41% indicated willingness to participate in the open trial and 24% to participate in the blinded study. When asked to take the perspective of an IRB, the three cohorts consistently favoured approving the open trial (83%) and, to a lesser extent, the blinded trial (54%). Study participants cited reasons for approving the hypothetical PCST consistent with those in the literature, such as methodological need, adequate informed consent, acceptable risk benefit ratio, and societal benefit outweighing the risks. Similarly, those who thought that an IRB should prohibit the PCST gave reasons including risk, lack of benefit to individuals, and expressed views that placebo-controlled trials are prima facie unethical [49].

These findings are consistent with Campbell et al. who asked patients with osteoarthritis about the acceptability of a PCST of knee arthroscopy [14, 17]. They found that many participants identified and supported the notion of a PCST (and some went on to consent to a formal pilot of a PCST), while others were sceptical of the need for a placebo. In discussing their own potential involvement in the trial, participants expressed a variety of views including a desire to help others and the hope that they would be allocated to the active rather than placebo arm of the trial. Others seemed to discount the possibility of harm from the placebo, or noted the potential inconvenience of being in a trial.

Thus research with relevant patient cohorts indicates that patient groups can be a rich source of information relevant to the development of PCSTs. The findings indicate qualified support for PCSTs and flag areas of misunderstanding about trial participation that should be addressed in the pre-consent educational materials that we advocate. There is no indication that the misconceptions of potential participants in PCSTs are greater than or differ from misconceptions experienced by participants in other types of clinical research. Importantly, the research we have reviewed does not support the view that patients are unable to deliberate about PCSTs. We therefore recommend the development of educational and decision-support materials should a PCST go ahead.

Our third suggestion concerns staff included in PCSTs such as anaesthetic, theatre and recovery staff, and those involved in any post-operative care (hereafter referred to as non-surgeon clinicians). Accounts of research ethics rightly focus on respecting and protecting participants, where respect is demonstrated through the process of informed consent [50]. One of the ethical justifications for informed consent is that it provides a mechanism for treating the participant as an end in him or herself, rather than treating him/her instrumentally, merely as a means to the researchers’ ends. Through the consent process, potential participants have the opportunity to accept or decline involvement in the research, and if they freely consent, concern about treating them instrumentally is alleviated. Other parties involved in the research, such as the clinicians who recruit patients or administer interventions, are not formally asked for their consent in the same way because they are not deemed to be vulnerable in the way that participants may be. They are usually the peers of the researcher and generally free to sign up to the protocol or to decline involvement with no adverse consequences. However, this approach is problematic in complex research interventions like surgery. Individual surgeons have the right to agree or decline participation in a PCST, but if they elect to participate, the nature of surgery means that their agreement necessarily commits non-surgeon clinicians to participation as de facto collaborators, because the surgery cannot proceed without them. If there is no explicit or formal process for seeking their agreement, then the surgeon’s commitment to be part of a PCST could appear to treat non-surgeon clinicians instrumentally as it does not accord them the respect of deciding for themselves whether or not to participate in the research.

This is especially problematic for ethically contentious research such as PCSTs. Participation in a PCST may expose non-surgeon clinicians to being deemed responsible for any harms that might occur during the trial, irrespective of whether or not they explicitly agreed to participate (e.g. an anaesthetic mishap during a placebo procedure). In the surgical setting, it is unclear as to what power non-surgeon clinicians have to refuse participation, and this may differ between institutions. Research with anaesthetists shows a range of views about a proposed PCST of knee arthroscopy [14]. As consultants and peers to surgeons, anaesthetists may well have the power to refuse to participate. However other non-surgeon clinicians, especially nursing staff, may find themselves committed by their surgeon (or anaesthetist) to a PCST about which they hold ethical reservations.

There are a number of issues here. First, participating in research is a legitimate part of the professional role of clinicians in publicly funded hospitals, so it might be considered unnecessary to seek agreement from all clinicians for all research. We agree that it is not unreasonable to expect research participation from clinicians; however, surgical research raises ethical questions in this regard. As we have noted above, non-surgeon clinicians lack the same power as surgeons in terms of participation in the research, as a surgeon’s commitment to a trial is understood to include the necessary surgical team to perform the research. Furthermore, this is not just a problem for individuals. While individual non-surgical clinicians may refuse, someone in the relevant role has to agree as there is a strong expectation that the research will go ahead with the requisite staff. This may be especially problematic if members of particular groups of non-surgeon clinicians tend to disagree about the importance and legitimacy of either the research question or the methodology of the proposed PCST. Next, the rewards of research may be unequally distributed. While chief investigators may receive recognition and advancement for their research activities and enjoy the creative aspects of research, these rewards may not be available to non-surgeon clinicians in the context of PCSTs where they are drawn in to other’s research activities. In addition, the ethically contested nature of PCSTs, the need for non-surgeon clinicians to engage in potentially deceptive behaviour in order to maintain the blinding, and the risk of being held responsible for patient harms during placebo procedures are all grounds for thinking that PCSTs are a special case which does warrant an explicit process for seeking agreement from staff who are necessarily involved just because it is a surgical trial.

Second, it may be that governance approvals are, or should be, the mechanism for dealing with these concerns. These typically occur after IRB approval, and are a site-specific examination of the governance and resource implications of the proposed research. It may be that this process will allow non-surgeon clinicians to voice their concerns, but if the concerns are primarily ethical rather than governance, they may be discounted on the grounds that the research already has ethics approval. Governance approvals do not ensure respect for the views and autonomy of participants in the way intended by formal consent processes mandated by IRBs.

Third, it may be impractical to seek explicit consent from every non-surgeon clinician affected by a PCST. It is already costly and difficult to mount surgical trials, such that adding a layer of consent maybe too burdensome and lead to less surgical research. This is a serious concern, yet research indicates that it is feasible to consult with those likely to be affected by a PCST [14]. Formalising this process would allow explicit consideration of the ethical concerns of non-surgeon clinicians.

In our view, non-surgeon clinicians who are necessarily involved in PCSTs should be recognised as relevantly vulnerable and their individual agreement should be sought prior to their inclusion in the research. This will require developing mechanisms for seeking agreement from non-surgeon clinicians to participate in PCSTs. Such agreement should be separate from governance approvals or feasibility assessments. Non-surgeon clinicians should be adequately informed about the proposed trial and then decide for themselves whether or not they are willing to participate. In addition, insurance arrangements for the trial should include cover for patient harms arising from placebo procedures in which non-surgical staff are implicated.

Our proposed expansions to the ethical review process for PCSTs are consistent with but extend beyond the principles promulgated by the American Medical Association regarding PCSTs [51]. The AMA principles engage with scientific validity and the conditions under which PCSTs may be justified, and the need for particular attention to informed consent, as do the works of other theorists [25]. The AMA principles stop short of mandating consultation with patient groups and the development of educational materials, and do not include non-surgeon clinicians as potentially vulnerable and therefore warranting the protection of formal agreement processes.

## Summary

Determining the ethical permissibility of PCSTs is complex. Here we have sought to establish that, given the nature and extent of surgical interventions and the existence of a surgical placebo effect, there is an in-principle justification for PCSTs to demonstrate efficacy, especially where risks of harm have been minimized, the outcomes are subjective, and there is no surgical comparator. In such situations, ethical consideration, rather than a blanket prohibition, is warranted. Ethical decision making about specific PCSTs can be supported by including the views of patient groups in deliberations about the risks and benefits of specific trials; developing educational materials to assist patient decision making; and including non-surgeon clinicians amongst those who should give formal agreement for participating in PCSTs. These measures will help to avoid unwarranted prohibitions of PCSTs on the part of IRBs, strengthen the decision-making of patient participants, and ensure that non-surgeon clinicians are not treated instrumentally. And while we have focused on PCSTs, a similar model may be applicable to other relevantly similar high risk research.

## Endnotes

^a^Placebo effects are improvements in a patient’s condition following an intervention that lacks known therapeutic mechanisms other than the patient’s belief in its value (Shapiro AK: **Factors contributing to the placebo effect. Their implications for psychotherapy.***Am J Psychother* 1964, **18**:73–88) [24].

^b^Some commentators argue that placebo controlled trials may be justified even when there is an active control available and no therapeutic effect is likely to follow from the placebo. Temple and Ellenberg, for example, note that because placebos control for non-specific effects such as the natural course of the disease or regression to the mean, they provide information that is not available in a trial with an active control. They argue that placebo controls are thus sometimes ethical even when an active control is available, but only in situations in which the patient will not be harmed by the delay incurred by treating with placebo. However, in the context of PCSTs, a therapeutic placebo effect may be expected, therefore placebos cannot fulfil the function they identify. (Temple R, and Ellenberg, S: **Placebo-Controlled Trials and Active-Control Trials in the Evaluation of New Treatments. Part 1: Ethical and Scientific Issues.***Ann Int Med* 2000, **133**(6):455–463).

^c^See for example, those summarized by Freeman et al. which include: the importance of the research question; lack of alternative methods that will provide equivalent data; methodological rigor including plausible mimic; indicative but not conclusive evidence that the intervention is effective; and an intervention that is sufficiently well developed to remain stable and current over the life of the trial (Freeman TB, Vawter DE, Leaverton PE, Godbold JH, Hauser RA, Goetz CG, Olanow CW: **Use of placebo surgery in controlled trials of a cellular-based therapy for Parkinson’s disease.***N Engl J Med* 1999, **341**(13):988–992, p.989) [3].

^d^It is worth noting that there are risks in both arms of the trial, placebo and active. There are no guarantees that the intervention will be effective or that the placebo will be more harmful than the intervention. For example, Miller notes that in a placebo-controlled trial of a laser myocardial revascularization device, there were no differences in outcomes between active treatment and placebo, but there were more adverse events in the intervention group (Miller FG: **The enduring legacy of sham-controlled trials of internal mammary artery ligation.***Prog Cardiovasc Dis* 2012, **55**:246–250) [36].

^e^See the information for families about getting involved at the Peninsula Cerebra Research Unit [http://www.pencru.org/].

## Authors’ information

WR is Professor of Clinical Ethics at Macquarie University, Sydney. Her background is in general practice and philosophy. She has a long-standing interest in research ethics and, as a member of the Australian Health Ethics Committee, has contributed to the development of national research ethics guidelines in Australia.

KH is a postdoctoral researcher at Macquarie University, Sydney. Her background is in philosophy. Her research focuses on the ethics of surgical innovation and surgical research, as well as theoretical questions about evidence based surgery.

ZCS is a research fellow at the Health Services Research Unit, University of Aberdeen, UK. Her background is in Sociology and her portfolio of research involves sociologically informed investigations of patients’ experiences of healthcare and of health services research. She has particular interest and expertise in using qualitative approaches to investigate patients’ perspectives of their care and treatment and also clinician – patient relations. She has had direct experience of exploring the acceptability of a placebo-surgical randomized trial of knee arthroscopy in the UK.

MKC is Professor of Health Services Research at the Health Services Research Unit, University of Aberdeen, Aberdeen, UK. Her background is in medical statistics and the design and methodology of clinical trials. She has long-standing interest in, and experience of, the design and conduct of clinical trials of surgical interventions. She has direct experience of exploring the acceptability and feasibility (including formal pilot) of a placebo-surgical randomized trial of knee arthroscopy in the UK.

## Abbreviations

IRB: Institutional Review Board
PCST: Placebo-controlled surgical trial
PD: Parkinson’s Disease.
